# Engineering *de novo* disulfide bond in bacterial α-type carbonic anhydrase for thermostable carbon sequestration

**DOI:** 10.1038/srep29322

**Published:** 2016-07-07

**Authors:** Byung Hoon Jo, Tae Yoon Park, Hyun June Park, Young Joo Yeon, Young Je Yoo, Hyung Joon Cha

**Affiliations:** 1 Department of Chemical Engineering, Pohang University of Science and Technology, Pohang 790-784, Korea; 2 Bio-Max Institute, Seoul National University, Seoul 151-742, Korea; 3 The Institute of Molecular Biology and Genetics, Seoul National University, Seoul 151-742, Korea; 4 School of Chemical and Biological Engineering, Seoul National University, Seoul 151-742, Korea

## Abstract

Exploiting carbonic anhydrase (CA), an enzyme that rapidly catalyzes carbon dioxide hydration, is an attractive biomimetic route for carbon sequestration due to its environmental compatibility and potential economic viability. However, the industrial applications of CA are strongly hampered by the unstable nature of enzymes. In this work, we introduced *in silico* designed, *de novo* disulfide bond in a bacterial α-type CA to enhance thermostability. Three variants were selected and expressed in *Escherichia coli* with an additional disulfide bridge. One of the variants showed great enhancement in terms of both kinetic and thermodynamic stabilities. This improvement could be attributed to the loss of conformational entropy of the unfolded state, showing increased rigidity. The variant showed an upward-shifted optimal temperature and appeared to be thermoactivated, which compensated for the lowered activity at 25 °C. Collectively, the variant constructed by the rapid and effective *de novo* disulfide engineering can be used as an efficient biocatalyst for carbon sequestration under high temperature conditions.

Carbonic anhydrase (CA) is an enzyme that rapidly catalyzes the hydration of carbon dioxide (CO_2_) into bicarbonate (HCO_3_^−^) and proton (H^+^)^1^. CAs are ubiquitous metalloenzymes that play various physiological functions in diverse forms of life and are classified into five different classes (i.e., α, β, γ, δ, and ζ)^2^. Due to the potential cost effectiveness and environmentally friendly properties, the use of CA in biomimetic CO_2_ sequestration is considered a promising alternative for reducing anthropogenic CO_2_, one of the most urgent environmental issues^3^,^4^,^5^,^6^. However, the application of CA has been limited due to the unstable nature of proteins, because CO_2_ reduction facilities generally operate under harsh environments such as high temperature conditions^6^,^7^. Thus, an improved thermostability of CA is a highly sought goal for successful industrial application.

Protein engineering has been widely applied to enzymes to increase thermostability. CAs have been engineered for use as stable biosensors or catalysts^3^,^8^,^9^,^10^,^11^,^12^,^13^. Although the directed evolution has proven effective for the given purpose^3^, it is generally a time-consuming and labor intensive process. Furthermore, the method requires a specific high-throughput selection/screening tool for each type of enzyme, whose implementation is greatly challenging and at times not feasible^14^,^15^. In contrast, rational design provides a fast, universal, and technically easier way to manipulate enzymes for desired traits. Although information on protein structure is an essential prerequisite for rational engineering, protein structure databases are fast-growing and design technologies are becoming more robust for better estimation^16^. In particular, disulfide engineering is a powerful strategy for enhancing protein thermostability. Various proteins have been successfully engineered by introducing disulfide bridges^15^,^17^,^18^,^19^,^20^,^21^,^22^. Disulfide bonds, including those found in naturally occurring proteins, are believed to decrease conformational entropy of unfolded state of protein, thereby providing folded state of protein with increased stability^17^.

A few studies have engineered CAs with disulfide bonds. Burton *et al*. developed a computational algorithm to stabilize human CA II (hCAII)^10^. The study showed the capability to predict potential residue pairs for disulfide engineering. However, the examined disulfide bonds were unfavorable, or the resultant stabilizing effects were not significant. In other studies, the naturally occurring disulfide bond from the unusually stable bacterial α-type CA of *Neisseria gonorrhoeae* (*ng*CA) was grafted into hCAII which has no native disulfide bond^8^,^12^. The engineered hCAII showed remarkable stability under high denaturant, high temperature or acidic conditions. However, this approach was fundamentally limited in that the engineering was restricted to the defined position where almost all bacterial α-type CAs already have a native, conserved disulfide bond^23^. Thus, a highly effective design and selection method is required as a more general strategy to introduce *de novo* disulfide bonds into CA enzymes.

In this work, based on flexibility analysis, novel disulfide bonds were successfully designed and engineered in *ng*CA as a model α-type CA to improve thermostability. In addition to thermostability, other biochemical properties of the constructed enzyme variants were characterized. We also provide a discussion for the possible factors affecting the kinetic and thermodynamic differences between the wild-type and variant CAs.

## Results and Discussion

### Design and selection of target residues for disulfide engineering

**Figure 1:**
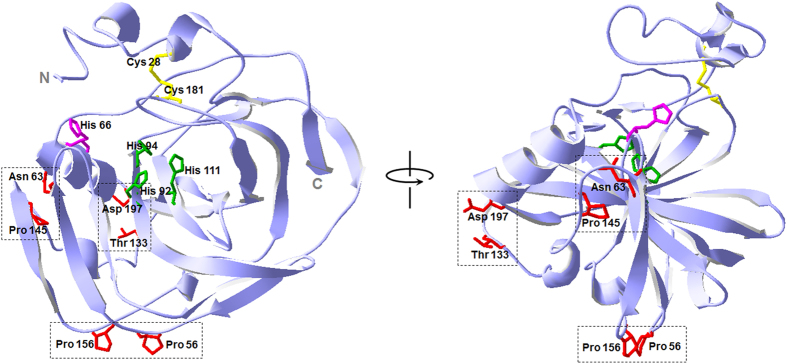
3D structure of *ng*CA and location of residue pairs for disulfide engineering. The ribbon model was constructed using Swiss-PdbViewer (http://www.expasy.org/spdbv). The structure was displayed in a front view (left) and in a side view (right) with an arbitrary axis. The residues for *de novo* disulfide engineering are shown in red. The residue pairs replaced with cysteines in each enzyme variant are boxed with dotted lines. The zinc (not shown)-coordinating histidine residues in the catalytic active site are shown in green. The proton shuttle histidine residue is in magenta. The native disulfide bond is colored yellow.

The *in silico* design of a novel disulfide bond was performed using Disulfide by Design 2 using the reported 3D structure of wild-type *ng*CA. This computational tool equipped with recently developed algorithms including protein fold recognition determines the residue pairs for potential disulfide bonds by evaluating both proximity and geometry of various residue pairs in the entire protein structure^24^. A total of 20 potential residue pairs, excluding overlapping pairs found in both chains of the homodimeric *ng*CA, were predicted to form disulfide bond when mutated to cysteines. It has been empirically suggested that newly inserted disulfide bridges from residue pairs with higher flexibility in a protein are more likely to be effective in thermostabilization of the protein^17^,^20^. Adopting this criterion, the list of residue pairs was ranked according to the sum of B-factor, i.e., a measure of residue pair flexibility^24^,^25^. Subsequently, three residue pairs with the highest B-factor values and the sufficient loop lengths (>25)^24^ were selected as experimental candidates for disulfide engineering (Table 1). These pairs appeared to be located on the surface of *ng*CA and far from the catalytic active sites, being considered permissive sites (Fig. 1).

### Expression of disulfide CA variants

**Figure 2:**
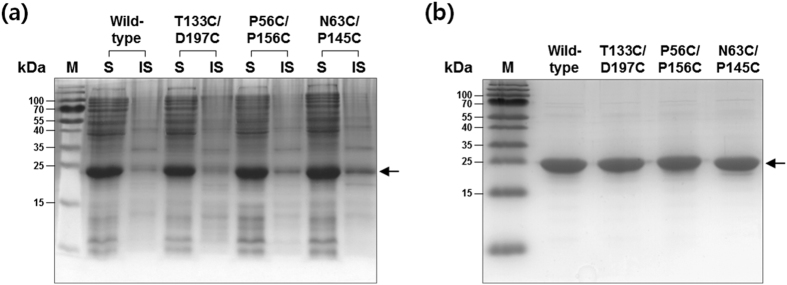
Expression and purification of disulfide CA variants. (**a**) Expression: cells were incubated at 25 °C after IPTG induction and fractionated into soluble and insoluble fractions. (**b**) Purification: each lane was loaded with 4 μg of each purified CA variant. The proteins were visualized with Coomassie blue staining after SDS-PAGE. The arrow indicates the position of the bands corresponding to *ng*CA variants. Lane: M, molecular weight marker; S, soluble fraction; IS, insoluble fraction.

Each candidate residue pair was replaced with cysteines by site-directed mutagenesis. The constructed three double mutants (T133C-D197C, P56C-P156C, and N63C-P145C) were expressed along with the wild-type *ng*CA in the SHuffle strain, an engineered *E. coli* strain that promotes cytoplasmic disulfide bond formation^26^. All enzymes were highly expressed in soluble forms with an average theoretical molecular mass of approximately 27 kDa (Fig. 2a). The enzymes with *C*-terminal His_6_-tags were purified to homogeneity (>97% purity as analyzed by ImageJ; http://imagej.nih.gov/ij)^27^ via one-step Ni^2+^ affinity chromatography (Fig. 2b).

Free thiol groups were quantified according to Ellman’s assay to verify the formation of the introduced disulfide bonds (Table 2). When the enzymes were treated with DTT, the number of free thiols in one molecule of wild-type *ng*CA was approximately 2, originating from the cysteine residues of the native disulfide bond (Cys^28^-Cys^181^) (Fig. 1), while the numbers in the variants were approximately 4, indicating that all of the selected residue pairs were successfully mutated to cysteines. In contrast, no countable free thiol was detected in any of the enzymes when DTT was not added, implying that all of the cysteine residues participated in disulfide bond formation. More direct evidence for the additional disulfide bond was provided by liquid chromatography-mass spectrometry (LC-MS) on N63C/P145C as a representative of the variants (See Supplementary Information). By peptide mass fingerprinting of tryptic digest of N63C/P145C, a peptide fragment with the linkage Cys^63^-Cys^145^ was identified as well as a fragment with Cys^28^-Cys^181^ (Supplementary Fig. S1), and this was further confirmed by peptide sequencing via MS/MS (Supplementary Fig. S2). Collectively, these results clearly indicate that the *de novo* disulfide bonds were successfully engineered as designed.

### Catalytic kinetics of disulfide CA variants

The enzymatic activities of the purified CA variants were measured at 25 °C using both *p*-NPA and CO_2_ as the substrates. Because CAs only in the α-class can catalyze ester hydrolysis^28^, the esterase activity has been widely exploited in the facile and reliable assays for α-type CA activity. The esterase activity of P56C/P156C was almost the same as that of the wild-type, whereas T133C/D197C and N63C/P145C showed an increased (149%) and a decreased (55%) activities when compared with that of the wild-type, respectively (Table 3). Although N63C/P145C showed the lowered activity, these results indicate that the structural stability of *ng*CA was not destroyed by the inserted disulfide bridges, which might otherwise lead to a complete abolishment of enzyme activity.

To obtain the kinetic parameters (*k*_cat_ and *K*_M_) in the CO_2_ hydration reaction, stopped-flow spectroscopy was used with *m*-cresol purple as the pH indicator. As shown in Table 3, the order of the relative *k*_cat_ values of the CA variants (T133C/D197C: 137%, P56C/P156C: 100%, and N63C/P145C: 19% of wild-type) corresponded well with the order of relative esterase activities despite a large discrepancy between the two activities of N63C/P145C. While this discrepancy has been reported in other studies, it remains unexplainable^10^,^12^,^23^,^29^. At first glance, the decrease in N63C/P145C activity seemed to result from an impaired proton transfer by the mutation, because Cys^63^-Cys^145^ is located near the proton shuttle residue (His 66) (Fig. 1). However, the *K*_M_ values were not significantly different from each other even in the case of N63C/P145C (Table 3). This means that the catalytic efficiency (*k*_cat_/*K*_M_) of N63C/P145C was lower than that of the wild-type. Because the lowered *k*_cat_/*K*_M_ is reflective of a slowed hydration step in the catalytic mechanism of CA^1^,^30^, the results imply that the CO_2_ hydration step in the active site, rather than the proton transfer from the active site, was the rate-limiting step in N63C/P145C when compared with that of the wild-type.

### Kinetic stabilities of disulfide CA variants

**Figure 3:**
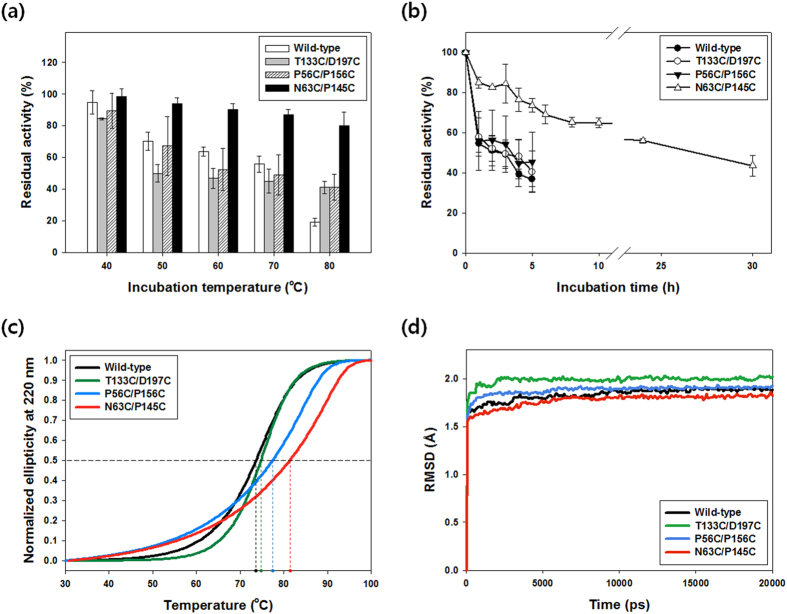
Thermostability of disulfide CA variants. (**a**) Short-term kinetic stability. The enzyme solutions (40 μM) were incubated for 30 min at different temperatures, and the residual activities were measured by esterase activity assay. Activities of 100% correspond to untreated samples. (**b**) Long-term kinetic stability at 70 °C. Each value represents the mean of at least three independent experiments, and the error bars represent the standard deviations. (**c**) Heat-induced denaturation of disulfide CA variants. Temperature-dependent changes of ellipticity were recorded at 220 nm on CD spectrometer. The denaturation curves were normalized to the fraction of unfolded protein. The horizontal dashed line indicates the point at which the fraction of unfolded protein is 0.5. The vertical dashed lines point to *T*_M_ values. (**d**) Overall RMSD of disulfide variants. MD simulation was performed at 400 K for 20 ns.

For the thermostability test, the CA variants were heat-treated under various temperature conditions from 40 °C to 80 °C for 30 min and the residual activities were evaluated compare to the activities of untreated (i.e., stored at 4 °C) enzymes. As shown in Fig. 3a, the enzymes other than N63C/P145C showed similar stabilities up to 70 °C at which approximately 50% of the initial activities were retained, while N63C/P145C showed a residual activity of 87%. At 80 °C, the stabilities of T133C/D197C and P56C/P156C were discriminated from that of the wild-type; the residual activities of the two variants were both 41%, whereas the wild-type retained only 19% of the initial activity. These results show that disulfide engineering successfully improved the thermostability of all three *ng*CA variants and N63C/P145C was exceptionally thermostable compared to the others.

To quantitatively compare the thermostabilities, the residual activities at 70 °C were measured in a time-dependent manner. As expected, the decrease in activity was drastic for all enzymes except N63C/P145C (Fig. 3b). N63C/P145C showed 56% residual activity after 24 h incubation, whereas the other enzymes lost more than half of their initial activities within 4 h. The half-lives (*t*_1/2_) of the CA variants were estimated by fitting the experimental data to an exponential decay curve. T133C/D197C and P56C/P156C had *t*_1/2_ of 4.7 h and 5.7 h at 70 °C, which were 18% and 43% longer than that of the wild-type (4.0 h), respectively. Notably, N63C/P145C showed a *t*_1/2_ of 31.4 h, exhibiting an approximately 8-fold increased stability compared with that of the wild-type. The enhancement was superior to those achieved in most of the other engineered enzymes by various strategies of rigidifying flexible sites^15^. It was also comparable to those found in the recent reports on the most successful disulfide engineering of industrially important enzymes α-amylase and lipase where 4.5- to 11-fold increases in *t*_1/2_ at specific temperatures have been demonstrated^18^,^20^,^22^.

### Thermodynamic stabilities of disulfide CA variants

To compare the thermodynamic stabilities, the heat-induced conformational changes of disulfide variants were monitored using a CD spectrometer at 220 nm. The mid-point transition temperatures (*T*_M_) where folded and unfolded proteins are in equilibrium were analyzed using the denaturation curves in Fig. 3c. The results show that T133C/D197C, P56C/P156C, and N63C/P145C have *T*_M_ values of 74.7 °C, 77.4 °C, and 81.4 °C, respectively, which were 1.1 °C, 3.8 °C, and 7.8 °C higher than that of the wild-type (Fig. 3c and Table 4). As expected, the order of the *T*_M_ values coincided with the order of the *t*_1/2_ values, implying that the denaturation of disulfide variants was the major factor that led to the inactivation of CA enzymes. The increase (7.8 °C) in *T*_M_ value of N63C/P145C was much higher than those of rationally engineered hCAII mutants with proline substitution (0.8 °C) or surface loop deletion (1.8 °C)^9^. In addition, the strategy of loop deletion for increasing surface compactness cannot be applied to bacterial α-type CAs, in which the surface loop regions are absent^23^. Thus, the experimental results demonstrate the general applicability of the *de novo* disulfide engineering as well as the effectiveness for rapid construction of highly stabilized enzyme variants.

We analyzed the contributions of thermodynamic parameters ΔH and ΔS to the observed conformational stability by calculations based on the van’t Hoff equation^31^. The free energy (ΔG) of protein unfolding can be defined as ΔH–TΔS where ΔH, ΔS, and T are enthalpy of unfolding, entropy of unfolding, and absolute temperature, respectively. Although all three disulfide CA variants showed increased stabilities as previously demonstrated, they were mainly attributed to different thermodynamic factors (Table 4). T133C/D197C had higher ΔH (stabilizing) and ΔS (destabilizing) of unfolding when compared to those of the wild-type, indicating that the enthalpic contribution was the major factor to the enhanced stability. In contrast, P56C/P156C and N63C/P145C exhibited lower ΔH (destabilizing) and ΔS (stabilizing), showing that the decreased entropic change of unfolding (i.e., the loss of conformational entropy of the unfolded state) by the disulfide bridge was the primary factor for the thermostabilization. These results are not surprising because design strategies aiming ‘entropic stabilization’ such as disulfide engineering do not always result in engineered proteins ideally with lower ΔS and unchanged ΔH^8^,^32^,^33^.

The above conceptual explanation can be readily verified in the denaturation curve (Fig. 3c). For instance, when compared to T133C/D197C, N63C/P145C showed faster increase in ellipticity in the relatively lower temperature region (<∼60 °C), implying that the denaturation of N63C/P145C can occur more easily in the lower temperature region due to the lower ΔH. However, as the temperature increases, the lower ΔS makes ΔG of N63C/P145C more slowly decrease. This is reflected in the gentle increase in ellipticity of N63C/P145C in the relatively higher temperature region (>∼60 °C) (Fig. 3c). In this respect, the improved stability of N63C/P145C can be more remarkable and effective than the other variants as temperature increases higher.

MD simulations were performed at 400 K to monitor the structural property of the disulfide variants at the molecular level^34^. As shown in Fig. 3d, the overall molecular root-mean-square deviation (RMSD) of N63C/P145C was the lowest among the others. This indicates that the introduction of Cys^63^-Cys^145^ improved the overall structural rigidity of CA enzyme. In addition, N63C/P145C generally showed lower residual RMSD compared to the wild-type (Supplementary Fig. S3). These results could be related to the above experimental data showing that N63C/P145C was the most thermostable variant. In addition, T133C/D197C showed the highest values in both the overall and the residual RMSD (Fig. 3d and Supplementary Fig. S3). This may explain and correlate with the increased activity of T133C/D197C (Table 3) and the increased ΔS of unfolding (Table 4).

### Temperature-dependent activities of disulfide CA variants

**Figure 4:**
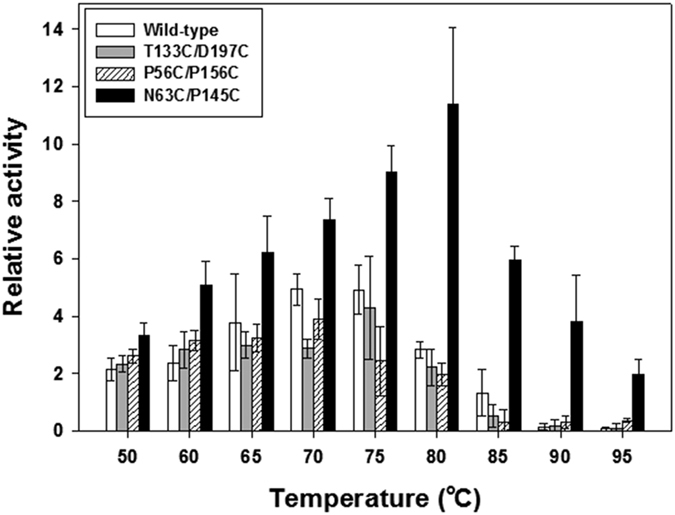
Effect of temperature on activity of disulfide CA variants. Esterase activities of disulfide variants were measured at each temperature and normalized to the activity of each enzyme at 25 °C. Each value represents the mean of three independent experiments, and the error bars represent the standard deviations.

Finally, we tested the effect of temperature on the enzyme activities of the disulfide variants by measuring esterase activities under various temperature conditions. As expected, the CA variants generally showed bell-shaped profiles for the temperature-dependent enzyme activity assay (Fig. 4). The wild-type and the other disulfide variants except for N63C/P145C showed optimal temperatures around 70 °C or 75 °C (Fig. 4). In contrast, N63C/P145C exhibited a continuous increase in enzyme activity until the temperature increased up to 80 °C. This type of upward shift in the optimal temperature is a generally observed effect of thermostabilization^35^,^36^,^37^. Interestingly, the extent of increase in N63C/P145C activity was significantly distinct from those of the others (Fig. 4). For instance, 1) N63C/P145C showed a 5.1-fold increase in activity at 60 °C compared with that at 25 °C, while the other enzymes had only 2.4- to 3.2-fold higher activities at the same temperature than those at 25 °C. 2) The maximal activity of N63C/P145C around its optimal temperature was 11.4-fold higher than the activity at 25 °C, whereas the others showed only 3.9- to 4.9-fold higher activities around their optimal temperatures than those at 25 °C. Although the wild-type, T133C/D197C, and P56C/P156C were more thermolabile than N63C/P145C under such high temperature conditions (Fig. 3a), the different extents of heat-induced damage during the measurement seemed to have only a minor effect on the observed differences in the extent of activity change because the enzymes were heat-exposed only for a short time (at most 30 s) during the activity measurement. Thus, the unexpected increase (thermoactivation) in N63C/P145C activity under the high temperature conditions was likely another effect of thermostabilization whose underlying mechanism is distinct from what caused the shift in optimal temperature^35^. This effect can beneficially compensate for the lowered activity of N63C/P145C at room temperature when compared with the wild-type (Table 3). Considering the shifted optimal temperature and the thermoactivation as well as the enhanced thermostability, the disulfide engineered α-type CA with Cys^63^-Cys^145^ can be a promising biocatalyst for efficient CO_2_ sequestration performed under high temperature conditions.

## Conclusions

Thermostabilization of CA is a major essential requirement for the industrial application of CA in biomimetic CO_2_ sequestration. In this study, we *de novo* designed and constructed disulfide engineered *ng*CA variants to improve thermostability. The design strategy was based on a flexibility analysis and was shown to be effective, generating a highly thermostabilized CA variant, N63C/P145C, out of the three constructed variants. The primary factor leading to the high thermostabilization of N63C/P145C was the decreased entropic change of unfolding with the increased rigidity. Although the activity of N63C/P145C was negatively affected by the inserted disulfide bond, the shifted optimal temperature and the thermoactivation along with the enhanced thermostability might enable the disulfide engineered CA to be a more efficient biocatalyst for CO_2_ sequestration under high temperature conditions.

## Methods

### Bacterial strains and culture conditions

All DNA work was performed using *Escherichia coli* TOP10 strain (Thermo Fisher Scientific, Waltham, MA, USA). *E. coli* SHuffle T7 Express strain (New England Biolabs, Ipswich, MA, USA) was used for protein expression. Cells were routinely grown in Luria-Bertani (LB) medium supplemented with appropriate antibiotics (50 μg/mL ampicillin for recombinant strains or 10 μg/mL streptomycin for wild-type TOP10 strain) at 37 °C and 220 rpm in a shaking incubator.

### Site-directed mutagenesis

Target residue pairs for potential disulfide bridges were designed and selected by the web-based program Disulfide by Design 2^24^ based on the three-dimensional (3D) structure of *ng*CA (Protein Data Bank (PDB) ID: 1KOQ). Residues were numbered according to the PDB sequence. Site-directed mutagenesis was performed by one step polymerase chain reaction (PCR)-based method as previously described^38^. The recombinant plasmid pET-ngCA encoding *ng*CA (NCBI GenBank accession number: Y11152) with a *C*-terminal hexahistidine (His_6_)-tag was used as the initial template DNA^39^. The codon encoding the first residue of a selected residue pair was first mutated, and the resulting plasmid was further used as the template for the mutagenesis of the second residue. The constructed plasmids were confirmed at each step by direct sequencing.

### Protein expression and purification

Recombinant *E. coli* SHuffle strain transformed with the constructed vectors was incubated at 37 °C. At mid-log phase (0.6–0.8 OD_600_), 1 mM of isopropyl-β-D-thiogalactopyranoside (IPTG; Carbosynth, Compton, Berkshire, UK) and 0.1 mM ZnSO_4_ (Samchun, Seoul, Korea) were added to the media for inducing protein expression. Subsequently, the cells were further cultivated for 8 h at 25 °C. After harvesting by centrifugation at 4 °C and 4,000 × *g* for 15 min, the cells were resuspended in lysis buffer (50 mM phosphate, 300 mM NaCl, and 10 mM imidazole; pH 8.0) and disrupted using an ultrasonic dismembrator (Sonics and Materials, Newtown, CT, USA) for 15 min at 20% amplitude on ice water. After centrifugation at 4 °C and 10,000 × *g* for 20 min, the collected pellet was designated the insoluble fraction (IS). The supernatant, designated the soluble fraction (S), was mixed with Ni^2+^ -nitrilotriacetic acid agarose beads (Qiagen, Valencia, CA, USA), and the recombinant CAs were purified according to the manufacturer’s instructions. The eluted proteins were finally dialysed against 20 mM Tris-SO_4_ buffer (pH 7.5) or 20 mM phosphate buffer (pH 7.5) at 4 °C. The concentration of the enzyme solution was adjusted to 40 μM.

### Protein quantification and analysis

Protein concentration was measured as previously described with slight modifications^40^. After the purified enzyme was denatured in 3 M guanidine hydrochloride (GuHCl)/0.1 M sodium phosphate buffer (pH 7.3), the absorbance of protein in the denaturing buffer was measured at 280 nm using a UV-Vis spectrophotometer (Shimadzu, Kyoto, Japan). Protein concentration was determined using the measured absorbance and the calculated extinction coefficient at 280 nm for each protein (34045 M^−1^ cm^−1^ for wild-type and 34170 M^−1^ cm^−1^ for mutants)^41^. Proteins were analyzed by sodium dodecyl sulfate-polyacrylamide gel electrophoresis (SDS-PAGE) followed by Coomassie blue (Bio-Rad, Hercules, CA, USA) staining.

### Quantification of disulfide bond

Free thiols were determined according to Ellman’s assay^42^. Denatured proteins in 3 M GuHCl/0.1 M sodium phosphate (pH 7.3) were incubated at room temperature for 30 min with or without 0.1 M dithiothreitol (DTT). Excess reducing agent was removed via repeated centrifugation using an ultrafiltration membrane with a 10 kDa molecularmass cut-off (Millipore, Bedford, MA, USA) and resuspension in the denaturing buffer inside an anaerobic chamber (Coy Laboratory Products, Grass Lake, MI, USA). Then, 10 μL of protein solution was added into a reaction solution containing 940 μL of the denaturing buffer and 50 μL of 2 mM 5,5′-dithiobis(2-nitrobenzoic acid) (DTNB; Sigma-Aldrich, St. Louis, MO, USA) dissolved in 0.1 M sodium phosphate (pH 7.3). After 5 min, the absorbance of the mixture at 412 nm was measured and the amount of free thiol groups was estimated using the extinction coefficient (13,600 M^−1^ cm^−1^) of 2-nitro-5-thiobenzoic acid (TNB).

### Esterase activity assay

Esterase activity assay was routinely used to assess relative activity of CA. 100 μL of 30 mM *p*-nitrophenyl acetate (*p*-NPA; Sigma-Aldrich) dissolved in acetonitrile was added to a cuvette containing 800 μL of buffer (20 mM Tris-sulfate; pH 7.5) and 100 μL of enzyme solution. The reaction was performed at 25 °C inside the spectrophotometer and the absorbance change at 405 nm was monitored for 3 min. The uncatalyzed rate of *p*-NPA hydrolysis was also measured by adding Tris-sulfate buffer instead of enzyme solution and subtracted from the observed data to obtain the enzyme-catalyzed rates. The enzyme activity was calculated using the extinction coefficient of *p*-nitrophenol (*p*-NP; 16,500 M^−1^ cm^−1^) at 405 nm. One unit (U) of esterase activity was defined as the activity that produces 1 μmol of *p*-NP per min.

### Steady-state kinetics

Initial rates of CO_2_ hydration were determined using a stopped-flow spectrometer (Applied Photophysics, Leatherhead, UK) by colorimetric method as previously described^43^. All preparations and reactions were conducted at 25 °C. 100 mM N-Tris(hydroxymethyl)methyl-3-aminopropanesulfonic acid (TAPS)/NaOH buffer supplemented with 57.2 mM Na_2_SO_4_ and 97 μM *m*-cresol purple (pH 8.5) containing the pre-dissolved enzyme was mixed with the same volume of CO_2_ solution in the stopped-flow cell, and the absorbance change was monitored at 578 nm. The CO_2_ solution with a concentration ranging from 5.63 mM to 33.8 mM (the saturated CO_2_ concentration at 25 °C) was prepared by diluting and mixing CO_2_-saturated water with N_2_-saturated water using a luer-lock syringe (HSW, Tuttlingen, Germany). Nine different CO_2_ concentrations were used. The uncatalyzed hydration reactions were also conducted for each CO_2_ concentration. Blank-corrected initial rates were used to determine the kinetic parameters (*k*_cat_ and *K*_M_) based on non-linear least squares fittings to the Michaelis-Menten equation using GraphPad Prism 6 (GraphPad Software, La Jolla, CA, USA).

### Thermostability test

Enzyme samples were aliquoted and incubated under appropriate temperature conditions in a water bath (Jeiotech, Daejeon, Korea). After removing aggregated proteins by centrifugation at 4 °C and 10,000× *g* for 10 min, the supernatants were stored at 4 °C until the enzymatic activities were measured using esterase activity assays. The residual activity (%) was calculated as the ratio of activity of heat treated enzyme to that of the untreated enzyme.

### Temperature-dependent enzyme activity

Temperature-dependent changes in CA activity were tested by measuring the esterase activity of CA at various temperatures (50 °C to 95 °C). The reactions were initiated by adding 100 μL of 30 mM *p*-NPA and 100 μL of enzyme solution in 20 mM phosphate buffer (pH 7.5) into 800 μL of preheated buffer (20 mM phosphate, pH 7.5) inside the spectrophotometer. The increases of absorbance at 405 nm were monitored for 20 s. At each temperature, the uncatalyzed rate of *p*-NPA hydrolysis was measured and subtracted from the observed data to obtain the enzyme-catalyzed rates. The obtained enzyme activities were normalized to the activities at 25 °C for each enzyme variant.

### Circular dichroism spectroscopy

Circular dichroism (CD) spectra were recorded on a CD spectrometer (Jasco, Tokyo, Japan). The CA variants at a concentration of 20 μM in 20 mM phosphate buffer (pH 7.5) were heated from 30 °C to 100 °C inside a quartz cuvette at a rate of 2 °C/min. The temperature dependent changes of ellipticity were monitored at 220 nm. The obtained curves were smoothed by non-linear regression using Sigmaplot 10.0 (Systat Software, San Jose, CA, USA). The mid-point temperature (*T*_M_), enthalpy (ΔH), and entropy (ΔS) of unfolding were analyzed according to a protocol by Greenfield^31^.

### Molecular dynamics simulation

All molecular dynamics (MD) simulations were performed by Discovery Studio 3.1 package (Accelrys, San Diego, CA, USA). The cysteine mutations and the disulfide bond conformation for each variant were manipulated by a macromolecule editing protocol. For additional simulations, CHARMm force field and Momany-Rone partial charges were used to assign atom types. The crystallographic water molecules were retained to mimic a hydration shell. The MD simulations were performed according to Standard Dynamics Cascade protocol. In detail, it consisted of 5 steps. The first and the second steps were the minimization steps. The maximum steps of both the steepest descent minimization method and the conjugated gradient minimization method were set to 20,000, and the RMS gradients were set to 0.1 and 0.0001, respectively. The heating step was set to 300 K for 100 ps. The equilibration and the production steps were 1 ns and 20 ns, and the temperatures for the corresponding steps were 300 K and 400 K, respectively. The production type was NVT. Implicit Solvent Model was applied, dielectric constant was set to 1, and implicit solvent dielectric constant was set to 80. The non-bond list radius was 14 Å. The trajectories of production steps were saved every 100 ps, and 200 frames were analyzed for comparison. For structural analysis, VMD 1.9.2 was used^44^.

## Additional Information

**How to cite this article**: Jo, B. H. *et al*. Engineering *de novo* disulfide bond in bacterial α-type carbonic anhydrase for thermostable carbon sequestration. *Sci. Rep.* **6**, 29322; doi: 10.1038/srep29322 (2016).

## Supplementary Material

Supplementary Information

## Figures and Tables

**Table 1:** Description of the double cysteine CA variants constructed in this study.

Variant designation	Position	Wild-type residues	>Loop length	Sum of B-factors
T133C/D197C	133, 197	Thr/Asp	63	87.60
P56C/P156C	56, 156	Pro/Pro	99	80.82
N63C/P145C	63, 145	Asn/Pro	81	77.17

**Table 2:** Quantification of disulfide bridges in the purified CA variants.

CA variant	Free thiol/protein (mol/mol)^a^		Deduced no. S-S bonds
	**−DTT**	**+DTT**	
Wild-type	0.06 ± 0.02	1.80 ± 0.14	1
T133C/D197C	0.08 ± 0.02	3.79 ± 0.22	2
P56C/P156C	0.06 ± 0.03	3.89 ± 0.06	2
N63C/P145C	0.08 ± 0.03	3.75 ± 0.05	2

^a^Numbers are represented in mean ± SD.

**Table 3:** Catalytic activities of the disulfide CA variants at 25 °C.

CA variant		CO_2_ hydration activity		
	**Relative esterase activity**^a^	***k***_**cat**_** × 10**^**−4**^ **(s**^**−1**^)	***K***_**M**_ **(mM)**	***k***_**cat**_**/*****K***_**M**_** × 10**^**−6**^**(M**^**−1**^ **s**^**−1**^)
Wild-type	1.00	1.44	14.2	1.01
T133C/D197C	1.49	1.97	16.7	1.18
P56C/P156C	1.03	1.44	16.9	0.85
N63C/P145C	0.55	0.27	17.3	0.16

^a^The specific activity of the wild-type corresponds to 0.22 U/μmol-enzyme.

**Table 4:** Thermodynamic parameters for protein unfolding.

CA variant	Melting temperature, *T*_M_ (°C)	Enthalpy change of unfolding, ΔH (kcal mol^−1^)	Entropy change of unfolding, ΔS (kcal mol^−1^ K^−1^)
Wild-type	73.6	48.8	0.141
T133C/D197C	74.7	52.8	0.153
P56C/P156C	77.4	35.1	0.091
N63C/P145C	81.4	30.0	0.085
