# Supplementary material for: Engineering de novo disulfide bond in bacterial α-type carbonic anhydrase for thermostable carbon sequestration
Source: Sci Rep. 2016 Jul 7;6:29322. doi: 10.1038/srep29322 (PMC4935852; doi:10.1038/srep29322)
Supplement: Supplementary Information [file srep29322-s1.doc]

**Supplementary Information**

**Engineering *de novo* disulfide bond in bacterial α-type carbonic anhydrase for thermostable carbon sequestration**

**Byung Hoon Jo,^a§^ Tae Yoon Park,^a§^ Hyun June Park,^b^ Young Joo Yeon,^c^ Young Je Yoo,^b,d^* and Hyung Joon Cha^a^***

^a^Department of Chemical Engineering, Pohang University of Science and Technology, Pohang 790-784, Korea

^b^Bio-Max Institute, ^c^Institute of Molecular Biology and Genetics, ^d^School of Chemical and Biological Engineering, Seoul National University, Seoul 151-742, Korea

§Authors equally contributed

*Correspondence to: H. J. Cha. Telephone: +82-54-279-2280; e-mail: hjcha@postech.ac.kr & Y. J. Yoo. Telephone: +82-2-534-2873; e-mail: yjyoo@snu.ac.kr

**SUPPLEMENTARY METHODS**

**Mass spectrometry.** Sample was prepared by digestion of 50 μg of N63C/P145C with modified porcine trypsin (Promega, Madison, WI, USA). Mass spectrometry (MS) was performed in positive ion mode at 200 μL/min on a quadrupole time-of-flight (QTOF) mass spectrometer (micrOTOF-Q III; Bruker, Billerica, MA, USA) coupled to a high-performance liquid chromatography (HPLC) (UltiMate 3000; Dionex, Sunnyvale, CA, USA). In HPLC, mobile phase A consisted of 0.2% formic acid in water and phase B consisted of 0.2% formic acid in acetonitrile. The tryptic peptide mixture was separated in analytical column (Acclaim RSLC 120 C18 2.2 µm 2.1×100 mm; Dionex) with a 2 min gradient ranging from 5% to 10% phase B, followed by a 41 min gradient from 10% to 30% phase B and a 7 minute gradient from 30% to 95% phase B at a constant flow rate of 200 μL/min. The information on the calculated masses of tryptic peptides was acquired using MassLynx 3.5 (Micromass, Manchester, UK). For tandem mass spectrometry (MS/MS), the four most intense precursor ions were selected for subsequent fragmentation using collision‐induced dissociation (CID). MS analyzer settings were as follows: capillary voltage, 4500 V; dry gas, 5.5 L/min; dry temperature, 180 °C; funnel 1RF, 400 Vpp; funnel 2RF, 400 Vpp; hexapole RF, 250 Vpp; quadruple ion energy, 5 eV; collision energy, 7 eV; collision RF, 600 Vpp; transfer time, 75 μs; pre-pulse storage, 8 μs; auto MS/MS, on; threshold for switching from MS to MS/MS mode, 5000 cts; active exclusion after 2 spectra for next 30 s. The MS spectra data were processed using DataAnalysis 4.0 and BioTools 3.2 (Bruker).

**SUPPLEMENTARY FIGURES**

**
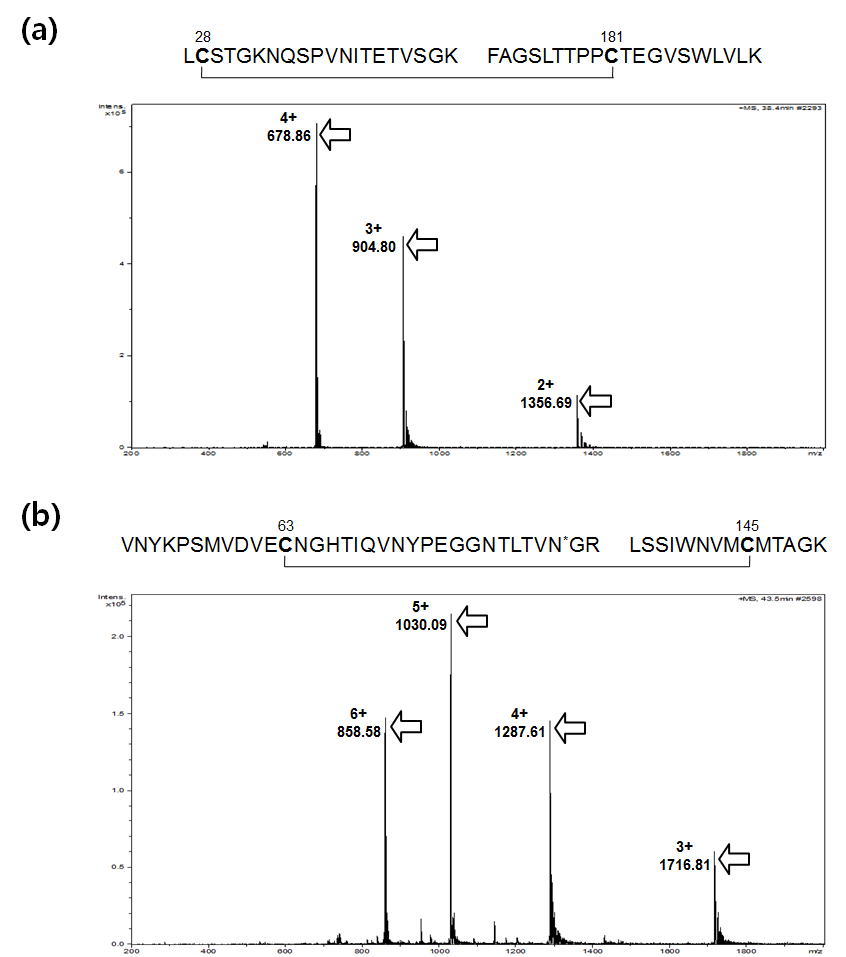
**

**FIGURE S1.** MS spectra of tryptic peptides of N63C/P145C with masses corresponding to the fragments with (a) Cys^28^-Cys^181^ linkage and (b) Cys^63^-Cys^145^ linkage. Above each spectrum, the amino acid sequences of the matched fragments are indicated along with the disulfide linkages. Asterisk (*) indicates the deamidation of the asparagine (N) residue.


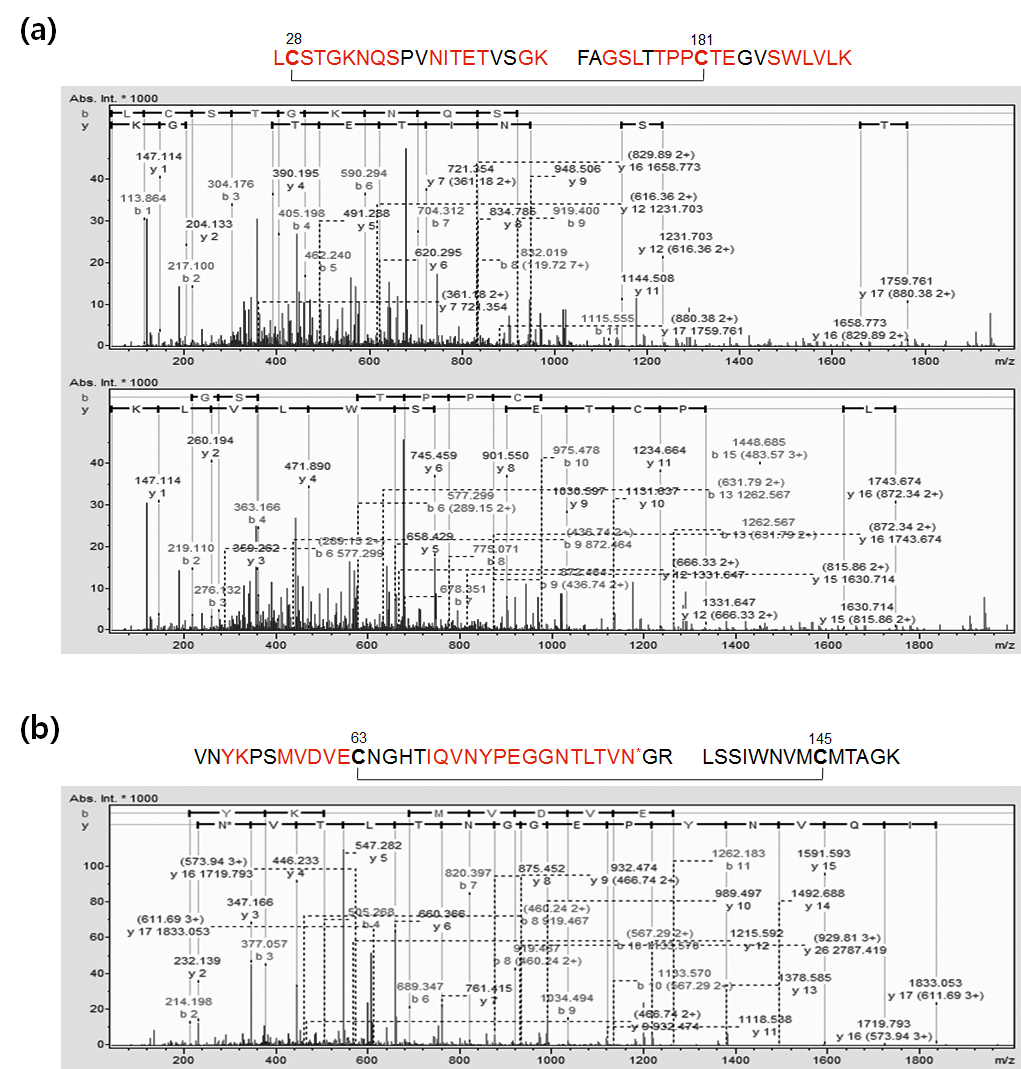


**FIGURE S2.** Peptide sequencing via MS/MS of the fragments with (a) Cys^28^-Cys^181^ linkage and (b) Cys^63^-Cys^145^ linkage. The amino acid residues identified by the analyses are highlighted in red. Asterisk (*) indicates the deamidation of the asparagine (N) residue.

**
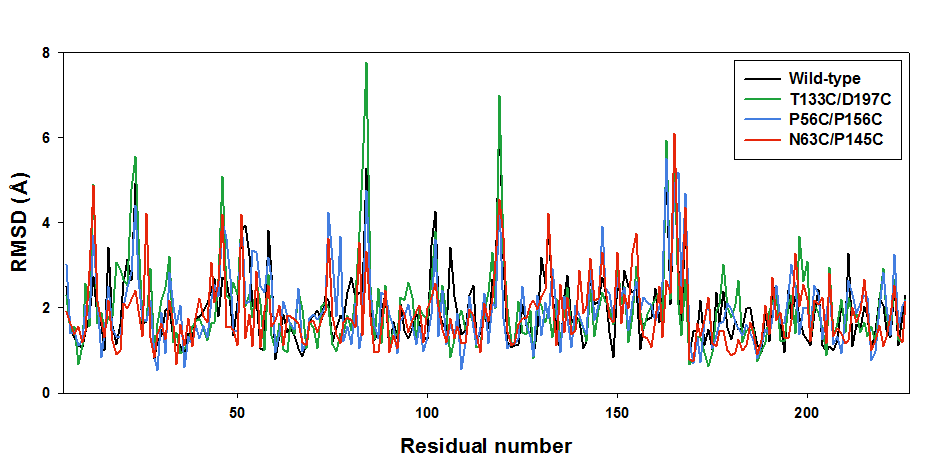
**

**FIGURE S3.** Residual RMSD of disulfide variants. MD simulation was performed at 400 K.
